# 

**Published:** 1859-02

**Authors:** 


No. IX. FEBRUARY—1859. Vol. XIV.

### BUFFALO MEDICAL JOURNAL AND Monthly Review OF MEDICAL AND SURGICAL SCIENCE.

AUSTIN FLINT, Jr., M. D.,
EDITOR.

BUFFALO, N. Y.
A. I. MATHEWS, PUBLISHER,
No. 220 Main Street.

Price $2.50, payable in advance, or $3.00 if not paid till the end of the year.

